# Rethinking solidarity towards equity in global health: African views

**DOI:** 10.1186/s12939-023-01830-9

**Published:** 2023-03-24

**Authors:** Caesar Alimsinya Atuire, Nicole Hassoun

**Affiliations:** 1 Department of Philosophy and Classics, University of Ghana, Legon, Accra, Ghana; 2 International Health and Tropical Medicine, University of Oxford, Oxford, UK; 3 Department of Philosophy, Binghamton University, Binghamton, NY USA

**Keywords:** Solidarity, Global health, Equity, Personhood, African philosophy, Faring Well, Fair allocation

## Abstract

When the COVID-19 pandemic first took the world by storm, the World Health Organization (WHO) issued a *Solidarity Call to Action to realize equitable global access to COVID-19 health technologies through pooling of knowledge, intellectual property and data*. At the dawn of 2022, 70% of rich countries’ populations were vaccinated but only 4.6% of poor countries (Our World In Data, Coronavirus (COVID-19) vaccinations, 2022). Vaccine nationalism and rampant self-interest grew and our ineffective global response led to new variants of concern - like Omicron - emerging. Rather than abandon the idea of solidarity in global health, we believe that the international community must embrace it. Solidarity, with its emphasis on relationality and recognition of similarities, could offer fertile ground for building an ethical framework for an interconnected and interdependent world. Such a framework would be better than a framework that focuses principally on individual entitlements. To defend this view, we draw on African relational views of personhood and morality. When humans are conceived of as essentially relational beings, solidarity occupies a central role in moral behaviour. We argue that part of the reason appeals to solidarity have failed may be traced to an inadequate conceptualization of solidarity. For as long as solidarity remains a beautiful notion, practiced voluntarily by generous and kindhearted persons, in a transient manner to respond to specific challenges, it will never be able to offer an adequate framework for addressing inequities in global health in a systematic and permanent way. Drawing on this understanding of solidarity, we propose pathways to respond creatively to the risks we face to ensure equitable access to essential health for all.

## Rethinking solidarity towards equity in global health^1^

### Introduction

Solidarity has gained traction in recent times. The concept has been invoked to motivate various national and international measures and initiatives to combat the COVID-19 pandemic. Yet, even though at local levels, especially in the initial phase of the pandemic, there were numerous gestures of solidarity, from a global health perspective, the calls to solidarity seem to have made little headway in the face of vaccine nationalism and other forms of protectionism. As Gavin Yamey put it, the world’s attempt to come together and distribute vaccines globally through COVAX, “was a beautiful idea, born out of solidarity… Unfortunately, it didn’t happen…Rich countries behaved worse than anyone’s worst nightmares” [1]. The behaviour of some richer countries, who by the way, also profess solidarity and adhere to initiatives like COVAX, has been described metaphorically by the African Union special envoy Strive Masiyiwa in the following terms: “Imagine we are in a village and there is drought and there will not be enough bread and the richest guys grabs the baker and they take control of the production of bread and we all have to go to those [rich] guys to ask for a loaf of bread” [2]. He added: “It’s not a question of if this was a moral failure, it was deliberate. Those with the resources pushed their way to the front of the queue and took control of their production assets” [3]. Solidarity, it seems, is turning out to be a weak driver for equity in the allocation of resources to combat a global pandemic like COVID-19.

Solidarity, a notion present in European and Catholic political discourse for over a century, is a somewhat late arrival in mainstream bioethical and global health discourse [4]. Bioethicists and global health experts are more at home with notions like justice, often understood from a liberal viewpoint as fairness, and concern for individual freedom. Some claim that these concepts, which generate negative and positive duties, lend themselves better to formulating enforceable legal frameworks that can hold people, organizations, and nations accountable. Solidarity on the contrary, often appears, “as a rather opaque term” [5].

Yet, despite these apparent conceptual weaknesses, there are historical examples of how solidarity has contributed to making important socio-political changes. The concept of black solidarity, expressed by authors like Frantz Fanon, William E. Du Bois, and Kwame Nkrumah, was important in the pan-African movement that accompanied the drive to end colonial rule in Africa [6]. In Poland, the fight against communist rule was led by the *Solidarnosc* movement. In the Czech Republic, Patočka appealed to a solidarity of the shaken to motivate resistance against Soviet dominion [7]. During the heat of the anti-apartheid struggles in South Africa, solidarity was often invoked as a motivation for African countries to support the cause led by the African National Congress [8]. In health and healthcare, successful national healthcare systems such as the UK’s National Health Service (NHS) can be described as grounded on a notion of solidarity [9].

The COVID-19 pandemic has underlined the ways in which everyone’s health is interdependent and interconnected. Whereas in past eras communities and populations could remain isolated or relatively independent from each other, today’s increasing interconnectedness means that our health depends on others’ health and also on the natural world given that many diseases (and likely SARS-CoV-2) are zoonotic. Added to this, the current challenges emerging from climate change, and environmental pollution and degradation, point to how our fates and the fate of the planet are all intertwined. Yet, interconnectedness and interdependence are only descriptive concepts that do not possess the normativity required to establish the equity needed for all to fare well and flourish. Historically, exploitation, injustice, and oppression have often been perpetuated along the fault lines of interconnectivity and interdependence. For example, the North Atlantic Slave Trade, and its accompanying racist agenda, were only made possible by a more interconnected world arising from the European explorations in the Americas and Sub-Saharan Africa in the late fifteenth century. For an interconnected world to be a fairer world, we believe an ethical framework capable of embracing all the interconnected parties is required [10]. So, as we become increasingly aware of our interconnectedness, there is a corresponding need for tools that can embed ethics into these relationships. We see solidarity, adequately conceived, as an attractive candidate for promoting fairness in an interconnected global health space.

Rather than abandon the idea of solidarity in global health as ineffective, we suggest taking a fresh look at the concept. This paper offers a conception of solidarity we hope will be useful in practice, though we do not defend the claim that we should have solidarity here and only aim to provide a new way of thinking about solidarity. We draw on African relational views of personhood and morality in articulating our proposal. In this view, humans are conceived of as essentially relational beings and solidarity occupies a central role in moral behaviour. Relationality and solidarity are not unique to African thinkers. Conceptualizations of humans as relational beings can be found in the Asian traditions as well as contemporary pro-communal thinkers in the Western tradition like Charles Taylor, Michael Sandel, and Alasdair MacIntyre. There is also a rich and long tradition of work on solidarity dating from Durkheim [11] to more recent works by Wiggins [12], and global health-focused works like those of Prainsack and Buyx [13], Gould [14], Jennings and Dawson [15], Dawson and Verweij [5], and Kolers among others.^2^ We focus on African conceptualizations because they add to the current discussion by arguing that solidarity is a primary ethical duty that arises from the way human beings are and ought to be rather than an instrumental or axiological means to assist those who are worse off. Secondly, appeals to solidarity during international health emergencies are often made in favour of assisting lower-income countries, most of which are found in Africa. Such international projects, like the ACT-Accelerator, bring together people and countries who may have different understandings of solidarity. One way to avoid talking past each other is to have a better understanding of each other’s conceptualizations and expectations. Understanding each other’s conceptualizations and expectations is a good starting point for defining shared actionable goals.^3^ We conclude by applying our conception of solidarity to evaluate the COVID-19 response and how we might better prepare for and respond to pandemics. If one endorses our view of solidarity, it should be clear that better pandemic preparation and response requires combatting artificially as well as naturally created scarcity; we must respond creatively to the risks we face to ensure equitable access to essential health for all.

### A conception of solidarity: an African perspective

The African view we present offers a framework in which practicing solidarity is an expression of being human; a view that we believe provides a new perspective, and that can shape the ongoing debate about what we owe to others. What follows considers first the notion of personhood within African philosophy and then a conception of moral personhood or dignity that draws on this account and grounds the conception of solidarity we advocate. By African conceptions we mean the views that are commonly expressed by African philosophical writers. This does not mean that such views are not present in other philosophical traditions, nor does it mean that all Africans subscribe to these views. The views we present are the salient ideas we find among the African philosophical writers. That is, we are drawing some common strands from diverse traditions that differ from one another in some important ways.

An initial iteration of our conception of solidarity is closest to Prainsack and Buyx [13] account in the bioethics literature. On their account, solidarity “comprises enacted commitments to carry costs – in the widest sense of the word – to assist others with whom a person or persons recognise similarity in a relevant respect”. They believe solidaristic “practices are enactments and expressions of who a person is, and wants to be” [13]. In the salient African philosophical traditions from which we will draw, however, solidarity is not just an “expression of who a person is, and *wants* to be” but an expression “of who a person is and *ought* to be” [13]. Thus, solidarity is not merely desireable or an ‘axiological value’ [13] but an expression of personhood. In this section, we look at the notion of (moral) personhood in African philosophy and the concept of solidarity that emerges from it.

#### Relatedness and moral obligation

One of the most cited texts in African philosophical reflections on personhood is Kenyan, Mbiti’s [16] claim that ‘I am, because we are; and since we are, therefore I am’. This is a cardinal point in the understanding of the African view of [hu]man” [16]. Within the ubuntu philosophical tradition, South African Ramose [17], similarly appeals to the saying that *motho ke motho ka batho; umuntu ngumuntu ngabanye bantu*, (a person is a person through other persons). Drawing from the Ghanaian Builsa—a Savannah ethnic group of West Africa—tradition, Atuire et al. [18], highlights how persons are defined as *nurbiik*, meaning a son or daughter of a person thus, inextricably relational. For the Builsa, a human being is considered as one who matters to someone [18]. Relationality would seem essential to the notion of personhood. Put another way, African philosophical traditions posit relationality as a given; personhood cannot be conceived of without relationality. Wiredu [19] underscores this position by recalling the Ghanaian Akan saying that *onipa firi soro besi a, obesi onipa kurom* (when a person descends from above, she descends into a human society).

Reflecting on these conceptions, Metz [20] teases out an Afro-relational conception of personhood. In his view, unlike Aristotle’s Categories, relations are essential to the definition of an object and persons, in particular: “the essence of any concrete, natural object is, at least in part, necessarily constituted by its relationship with elements of the world beyond the thing’s intrinsic properties” [20]. He posits this against “the Anglo-American, and more broadly Western, philosophical tradition,” where “the self or person is usually identified with something internal, either a soul that contains mental states, a brain that contains mental states or, most common these days, a chain of mental states themselves, some of which are self-aware” ([20], p. 215). Metz’ account echoes Nigerian Menkiti’s [21] claim that whereas most Western views of human “abstract this or that feature of the lone individual and then proceed to make it the defining or essential characteristic which entities aspiring to the description ‘[hu]man’ must have, the African view of [hu]man denies that persons can be defined by focusing on this or that physical or psychological characteristic of the lone individual”. What emerges from these various positions is a relational view of personhood whereby persons emerge out of a web of relations.

Moreover, drawing on the views of Akan philosophers like Wiredu and Gyekye, who see these relations as extending to all humans, the conception of solidarity in the context of health crises like international pandemics, we suggest that *all* humans are part of one global community. That is, the conception of solidarity we offer is properly cosmopolitan. We start from a conception of our relationships to others in a theory of moral personhood and claim that these relationships require us to sympathetically put ourselves in each person’s shoes to stand in solidarity with them and collaborate creatively to ensure that we all fare well enough together. There are, of course, other possible ways of thinking about solidarity but we explain in the paper why we prefer this one to some of the main contenders in the literature on bioethics.

#### A normative conception of personhood

African concepts of morality emerge against this backdrop of what it means to be a person. Given that persons are relational beings, morality is that which creates and fosters the conditions necessary for members of and the collective to be able to fare well enough. Thus, according to Wiredu [22], ‘a certain amount of altruism is absolutely essential to the moral motivation’. As Mbiti put it, “only in terms of other people does the individual become conscious of his own being, his own duties” the act of becoming conscious of one’s personhood entails an assumption of duties [16]. The fulfillment of these duties, which are mostly other regarding virtues, is what constitutes the moral notion of personhood.

African theorizations of personhood focus more on the moral notion of personhood. Thus, on some accounts, personhood becomes a human ideal to be achieved. Menkiti ([21], p. 172), describes moral personhood as “something which has to be achieved, and is not given simply because one is born of human seed”. Also, Gyekye [23], makes the claim that personhood is typically “earned in the ethical arena: it is an individual’s moral achievement that earns him the status of a person. Every individual is capable of becoming a person inasmuch as he is capable of doing good and should therefore be treated (potentially) as a morally responsible agent”. Against the objection that some humans may be incapable of becoming fully morally responsible agents, Gyekye suggests that such we still owe duties such individuals because they are humans. Atuire [24] draws on the concept of a privation to say that cognitive or psychological impairments that limit the capacity to become a fully morally responsible are accidental and do not alter the nature of a being, thus duties owed to all humans are also owed to such persons. What is more, a truly relational ethic that prioritizes the faring well of all points to doing more to assist those whose capacities are limited.

On this view, Molefe [25], affirms: “Put simply, to be a good human being I am required to exercise my duties to others. The best way to focus on what is morally best for me as an agent is to focus on bettering the humanity of others”.

The main thrust of African ethics according to Wiredu ([19], p. 194), “would seem generally to be of a humanistic orientation”. This, he continues, is sustained by the fact that the most common formulation of morality centres around the expression, *onipa na ohia* (it is a human being that has value). The saying can be interpreted both as “all values derive from human interests’ and ‘human fellowship is the most important of all human needs” [19]. Wiredu proposes a moral imperative: “In all inter-personal situations put yourself into the skin of the other and see if you can contemplate the consequences of your proposed action with equanimity” ([19], p. 198).

The notion of solidarity we outline below is grounded on this moral framework in which to be a person means to be-in-relation and to be moral means promoting one’s own well-being by ensuring that others also fare well enough. Thus the Akan saying, “If you do not allow your neighbour to reach nine you will never reach ten” [23].

### Defining solidarity

Drawing on the African frameworks that we have set out, we propose the following definition of solidarity, broadly: *a sympathetic and imaginative enactment of collaborative measures to enhance our given or acquired relatedness so that together we fare well enough.* The key to understanding what this kind of solidarity requires, then, is understanding our given interrelatedness and how we can live well enough together, but first we must explain how we should sympathetically and imaginatively collaborate to achieve this goal.

#### Sympathetic

Solidarity, as Prainsack and Buyx point out, entails a recognition of similarity with others in a relevant aspect. It does not, however, stop at a recognition of affinity, it requires sympathy (*syn*--with, together and *pathos*--passion, suffering, emotion, feeling) [13]. Sympathy entails recognizing, and to some extent identifying with the other. In the present case of the COVID-19 pandemic, assisting low- and middle- income countries will thus not be motivated by the fear that if persons from economically poorer countries remain unvaccinated, new variants can appear that will render the vaccinations in the high-income countries inefficient. Such a view is ultimately about protecting oneself within a world of inextricable global interdependence. It simply sees the well-being of others as instrumental and may even allow for forms of vaccine nationalism when the interconnectedness becomes unnecessary or of little value. Solidarity points more in the direction of seeing the other as another self whose well-being is conjoined to our own. We empathize with others putting ourselves into their shoes in deciding what they need to flourish, in part, because their flourishing is part of our own [26, 27].

#### Imaginative

By imaginative we mean that solidaristic initiatives often go beyond the established channels of social welfare to find paths to assist others. Often solidarity requires *creative resolve -* a fundamental commitment to overcoming apparent tragedy [28]. Solidarity can be institutionalized and even codified into legal mechanisms as we see in national healthcare systems where all are expected to contribute to the health needs of fellow citizens through taxation. Institutionalized solidarity still leaves room for other forms of solidarity to be practiced at interpersonal and community levels. These latter forms which can be spontaneous, or transient, and often require going beyond established channels to assist others, or each other, in achieving a desired goal. Examples of such forms of solidarity during the COVID-19 pandemic include shopping for members of the community who are in isolation, clapping for healthcare and frontline workers at an established time and day, and many other gestures [28]. Nevertheless, the imaginative and often spontaneous character of solidaristic initiatives do not make solidarity optional or supererogatory. The normative requirement is to find paths to assist so that we all fare well enough together.^4^ Where there are no established routes or when the existing channels are inadequate or inefficient, it takes imagination to find novel pathways.

#### Enactment of collaborative measures

Sympathy and imagination alone do not suffice to establish solidarity. Solidarity is an action orienting concept, thus, feelings or attitudes need to be transformed into real measures or gestures for solidarity to occur. Feeling sympathy for members of marginalized communities in India or Brazil who have little access to COVID-19 tools does not become solidarity until these feelings are transformed into concrete actions geared towards ameliorating their condition.

A common characteristic of the creative and imaginative dimension of solidarity is the capacity to generate new groups or new forms of relationships within existing groups to respond to challenges. When this happens, the common goal of the group becomes what Taylor [29] calls an executive goal. For example, a group of travellers on a train that breaks down before arriving at a destination may establish a solidaristic group to assist each other to arrive at the desired destination by, say, coming together to hire a bus or some other means of transport. Such a group may even assist travellers who need physical or economic help. However, once the executive goal of arriving at the destination has been achieved, the group may cease to exist as a solidaristic group. For other types of executive goals, say healthcare or worker’s rights, solidaristic groups can, over time, become institutionalized. When this happens, as the initial trigger that catalyzed the establishment of the group recedes in individual and collective memory, solidarity can feel imposed or coerced if motivations are not constantly renewed. This happens especially in large solidaristic groups like nationwide healthcare systems or social welfare programmes.

#### Enhance intrinsic and acquired relatedness

Whereas solidarity can be borne out of transient conditions like a train breaking down, there are other types of solidaristic groups that are born out of the intrinsic human relatedness that we have described above. This type of solidaristic exigency is not transient; it is a permanent requirement for human flourishing. Arguably, health and access to healthcare fall under this exigency since they are important for flourishing. The specific modalities or institutions that are created as a response to the solidaristic exigency can change and need to adapt to be better attuned to achieving the desired goals. What is constant is the requirement of solidarity as a framework in which relatedness is not just a descriptive reality but one that is a normative requirement to ensure fairness, equity, generosity and compassion. It is a moral obligation because it makes us more human as moral persons. And, becoming more human, in the African view, which we take as the starting point for our argument, is a moral duty. Solidarity embraces justice because it ensures that we fulfill our duties towards others by recognizing our common humanity that is grounded, in part, in the relationships we stand in to others.

#### Faring well enough together

The notion of faring well enough together is grounded on the idea of our human interrelatedness. Faring well thus becomes both an individual and communitarian enterprise. Ideally, to fare well together requires imagination and sympathy to put ourselves into others shoes and consider whether we would be content to live their life. As reasonable, caring, and free people we ask ourselves whether there are any serious reasons to doubt the person can live their life well enough considering their history, psychology, social relationships, values and so forth. We should empathetically consider whether we would be content living another’s life in order to reach a judgment about whether their lives are sufficiently good [27]. We believe that reasonable, caring and free people should put themselves in others’ shoes in figuring out what makes a life minimally good. Resolving disagreements about what people need to live minimally well may require deliberation and discussion with others who are similarly reasonable, caring and free. Whatever form the minimally good life takes, this account of solidarity sets the following standard: When a person’s ability to live a minimally good life is not secure, then she is entitled to the aid of others [26, 27] in helping her secure a minimally good life, provided that this help will not jeopardize their own ability to live a minimally good life [26, 27]. The notion of solidarity that we are putting forward here leads us to think that the human condition is such that solidarity is a necessary ingredient for a moral community. Humans, as relational beings thrive morally when they strive to make other persons thrive.

### African conceptions of health and ill-health

To explore the relationship between health and the notion of solidarity we are presenting, we need to examine the African conception of health and healthcare. A common view in many African frameworks is that good health is the outcome of a harmonious combination of physical, non-physical, and social factors. Health, as Omonzejele [30], puts it:

is not just about the proper functioning of bodily organs. Good health for the African consists of mental, physical, spiritual, and emotional stability of oneself, family members, and community; this integrated view of health is based on the African unitary view of reality. Good health for the African is not a subjective affair [30].

Gbadegesin [31], drawing from the Nigerian Yoruba tradition, says:

The Yoruba, like most African culture groups, have a holistic conception of health and disease. To be well or healthy is to be in a position to do one’s daily tasks; it is to have a strong body and mind. (….) the Yoruba word for health, *alaafia*, means more than physical health. It refers to a person’s physical, social, psychological and spiritual well-being. If any of these aspects of a human’s life is in a state of disease, then she cannot claim to have *alaafia*. (…) A person who is not healthy or who is ill is in a state of dis-ease and needs to be reinstated wholly [31].

If we focus our attention on the holistic and relational view of health put forward by Omonzejele and the notion of disease by Gbadegesin as a condition of being ill-at-ease, we begin to understand why it is common in Africa for persons to recur to both biomedical cures and other forms of metaphysical practices to seek healing. The underlying framework is that the individual’s health is linked to other factors that derive from the relationality of being human. Although this vision in Africa is often clouded with religious or ritualistic connotations, from a philosophical viewpoint, it is not far removed from emerging concepts in global health that insist on socio-cultural determinants of health and notions like intersectionality [32].

From this perspective health is both an individual and a shared good. Thus, the pursuit of individual health requires not only cultivating one’s physical well-being but ensuring the wellness of the relations (human, environmental and other) that are part of this broader notion of health. In the specific cases of epidemics and pandemics, such vast disruptions of health are seen as a common threat that cannot be overcome without the collaboration of all. What is more, the solution does not lie only in curbing the spread of the disease (effective vaccines or therapeutics), but also seeking to establish what behaviours may have contributed to the emergence and spread of the disease. A way to characterize this, as Jecker and Atuire [4] suggest, is to see an event like the COVID-19 outbreak not as a pandemic, but a *syndemic,* − “convergence of biosocial forces that interact with one another to produce and exacerbate clinical disease and prognosis”. Focusing the ‘syn’ (with, together) enables us to understand the emergence and spread of disease as the coming together of various factors. And this in turn informs a response that requires the coming together of persons and tools. Fighting a syndemic or widespread disease ultimately entails seeking greater harmony between persons collectively and ensuring more harmonious relationships with the environment. The predominant conception of health in many African cultures, then, is in line with the WHO’s definition on which “Health is a state of complete physical, mental and social well-being and not merely the absence of disease or infirmity.” Some will object that people can fare poorly for reasons that have nothing to do with health, arguing that, for instance, a broken heart may not qualify as a health problem or that some are lazy, poor, sad, wicked or otherwise poorly off, while perfectly healthy. Yet, even those who reject this definition of health, should agree that people need the health-related components of welfare, whatever those are exactly, to fare well enough. So, one can adopt a pragmatic, functional, or normative conception of health and still endorse our conclusions (though the scope of what solidarity requires may change slightly depending on one’s account, for example sympathy may not count as important). It only matters that health is often an important component of, or precondition for, faring well enough.

From our view, and others, the pursuit of health necessarily requires solidarity: *a sympathetic and imaginative enactment of collaborative measures to enhance our given or acquired relatedness so that together we fare well enough.* Moreover, we should stand in solidarity with others because we are related to them (and our flourishing together depends on it) and to do so we must empathize with them appropriately (so we in fact flourish together).

### Alternative conceptions of solidarity in bioethics

To defend our conception of solidarity (and the framework based on it), however, it is important to explain how it compares with and complements the main alternatives in the literature on global health and bioethics. Conceptions of solidarity in global health and bioethics can be grouped into three models or approaches.

#### The axiological model

This model sees solidarity as axiological; that is something linked to goodness, ideals, and virtues. Axiological models tend to focus more on moral agents and how they ought to behave whereas deontic models pay more attention to the actions. For Heyd [33] “the deontic sphere of morality is often taken as describing the *minimal* conditions of morality, the basic requirements of social morality that secure a just society, while the axiological sphere aims at higher ideals which can only be commended and recommended but not strictly required. In its deontic nature, morality is closely associated with the legal, while the axiological is closer to the ideal or the ideological (sometimes referred to as “the ethical”).”

Prainsack and Buyx ([13], p. 79), follow this model when they assert that, “while justice is a thoroughly deontic – and more so, a universal – principle, solidarity is the ‘putty’ that fills some of the gaps that justice leaves open, for inter-individual, prosocial and supererogatory behaviour”. Thus, solidarity would seem to be a bottom up approach to achieving decent and just societies. However, at the institutional level, they hold that “justice is destined to guide the creation of mechanisms for the allocation of resources in the widest sense of the word, ranging from the distribution of tax burdens to healthcare to the right to vote, often enforced by state or regional forms of power” (p. 45). This is because solidarity “cannot be mandated and sanctioned in the way duties of justice can”.

The axiological model, which seems to be the most common understanding within global health discourse, in our opinion limits solidarity to an exercise of supererogation ([13], p. 79). Thus conceived, solidarity is contrasted with justice which is seen to be deontic, sanctionable and actionable at institutional levels. Solidarity may thus seem to be an optional activity that good and praiseworthy persons and institutions engage in. In the context of the COVID-19 pandemic, such a view perhaps explains why high-income countries can practice solidarity by adhering and contributing to the ACT-Accelerator and COVAX whilst at the same time practicing vaccine nationalism. Standing in solidarity with low-and-middle-income countries is perceived as an act of generosity, a good thing to do, not sanctionable, and supererogatory.

This view differs radically from ours because we see solidarity as morally foundational. Rather than a ‘putty’, it is the basic building element, call it the cement, out of which the building blocks of a just society can be molded. If solidarity is not sanctionable because it seems too idealistic or too tall a call, we need to keep in mind that the same can be said about the ideal of justice. Yet, through centuries of effort, humanity has been able to elaborate tools (and sanctions) for building societies that try to practice justice. In other words, we can, if we want to, build tools to enhance solidarity, and also sanctions for failures to exhibit solidarity. We may need to think about sanctions in a way that is different to those we currently apply for failures in living up to justice.

#### The instrumental approach

Gould [14] offers an “alternative reading of solidarity in healthcare drawing on social movement and labor contexts” that highlights “a crucial dimension of contemporary healthcare provision, namely, structural injustice. Systemic forms of injustice militate against adequate healthcare for all, and suggest the need for solidaristic action to struggle against and to remedy existing entrenched inequalities” ([14], p. 2). In her view, solidarity is an effective way to address and overcome the structural injustices that underlie issues of social concern like health. Thus, whilst admitting that solidarity could be required for its own sake, what is important for bioethics is a view in which solidarity is aimed at promoting shared interests and overcoming domination and exploitation with the aim of achieving justice and equity. In her view, even though this form of solidarity is usually found among groups that share a similar situation of injustice (unity solidarity), such groups can also form a global network of solidarity, “[coming together across borders to fight for social justice and] to help alleviate suffering” [14]. This form of networking solidarity is apt for capturing constructive relations across borders and towards distantly situated others. This view focuses on solidarity as an effective tool in the fight against societal injustices. Thus, ideally, in the absence of such structural injustices, solidarity might not be needed at all.

Gould’s view is a reactionary approach to injustice. For Gould, solidarity has a restorative power to push for justice in the face of societal and structural injustice. This view differs from ours because, even though it acknowledges solidarity as a value in itself, it focuses on the instrumentality of solidarity in achieving justice. Our view is that, if solidarity were practiced in the first place, the injustices that Gould seeks to combat through solidarity would not exist. Rather than a reaction to injustice, solidarity as we see it, is foundational and necessary for building societies in which we contribute to each other’s flourishing. Injustice, especially structural, is ultimately a breach of solidarity. Thus, rather than appeal to solidarity as a tool for fighting injustice, we see the implementation of solidarity as a means to preventing injustice.

#### Relational approaches

Finally, Dawson and Jenning [34] and Tosam et al. [35] offer relational accounts of solidarity that are in many ways similar to ours. Tosam et al. draw from African ubuntu philosophical views of personhood to account for a solidaristic approach to global public health [35]. For them an “African approach to solidarity stems from the African conception of a person as an interdependent being [35]. As interdependent persons with a common destiny, individual persons and communities have the responsibility to share with and protect one another” ([35], p. 246). It is not only African accounts that see solidarity as a foundational dimension of human relationality. Dawson and Jenning [34], using a semantic approach, arrive at a conception of solidarity that is foundational and relational. For them, solidarity “arises from the nature of humans as biological and social creatures. It is a constitutive concept, not a voluntarist one.” ([34], p. 76). This, in their view, ought to lead us to a view of solidarity as a “deep and enmeshed concept, a value that supports and structures the way we in fact do and ought to see other kinds of moral considerations” ([34], p. 73-74). Thus, solidarity for them is not a value to be contrasted with other or “added to any list of values”.

An important aspect that these views on solidarity highlight, which we see as enriching and perfecting the African conceptualization that we have outlined, is the insistence on justice. This is relevant because not all solidaristic groups are always focused on justice and equity for all. In fact, some mafia-type groups like the *ndrangheta* and the *camorra* in Southern Italy are infamously solidaristic among members. Yet, their goals and actions can hardly be described as pursuing justice for all. Solidarity grounded on the African notion of faring well together as humans implicitly requires equity and justice for all. Highlighting the relationship between justice and solidarity makes this even more explicit, thus preempting the risk of limiting solidarity to only those we can easily empathize with, which would be a form of tribalism.

### Why solidarity requires creative resolve in protecting the vulnerable and investing in basic health systems

As we explain below, solidarity on our account, assumes that human lives everywhere have the same value and that a public health emergency like COVID-19 is a threat to the lives and livelihoods of all humans. We subscribe to the often-repeated phrase that, in the context of the pandemic, no one is truly safe until we are all safe. This phrase that has been repeated by various leaders supports protecting vulnerable people everywhere and not just those in rich countries, and this requires helping everyone access the existing resources they need to prepare for and respond to terrible diseases like COVID-19, but also addressing the issue of often artificially induced medical resource scarcity.

In our solidaristic framework, resources should be distributed in line with needs, not economic power, because global health is a global public good. So absent evidence that a different distribution will better help everyone live at least minimally well, allocation should be based on proportions of vulnerable populations. Globally, persons most at risk of dying or suffering severe disability if infected should receive priority attention. However, the relevant resources do not just include vaccines but preventative equipment (PPE etc), diagnostics, therapeutics (monoclonal antibodies, etc) and other essential health protecting technologies.

Moreover, an important part of solidarity is to overcome the fundamental problems of scarcity. That is, helping the vulnerable often requires what one of us has termed *creative resolve:* a fundamental commitment to overcoming apparent tragedy [28]. To have creative resolve, we must not only question evidence against the possibility of helping the vulnerable but come up with creative ways of doing so and act to help them insofar as possible and otherwise permissible. It is not enough to consider all the options on the table, we must put new ones on that table.

Consider a few examples from the history of public health of how people have successfully responded to failures of solidarity to restore relationships with creative resolve. By the late 1990’s, most people in rich countries were living long and productive lives with HIV/AIDS due to antiretroviral drugs. At the same time, millions of people in poor countries were still dying in droves from lack of access. South Africa’s Treatment Action Campaign refused to accept pharmaceutical companies’ claim that it was impossible to lower prices from US$12,000/per patient per year. They educated patients to demand access to treatment and engaged in legal battles that eventually forced prices down to approximately US$350/per patient per year [28, 36].

Or consider how Agnes Binagwaho helped increase life expectancy in Rwanda from 27 years to 65 years [37]. She worked with the Rwandan Ministry of Health to reduce the incidence of non-communicable diseases as well as HIV/AIDS. She refused to accept the going wisdom that fighting cancer was not cost-effective in poor countries like Rwanda. Instead, she decided to create a human papillomavirus infection (HPV) vaccination campaign for girls to prevent cervical cancer [37]. During her time as leader of Rwanda’s Nationals AIDS Control Commission, she similarly expanded efforts to fight HIV/AIDS and reduced AIDS-related deaths 44% [38].

Finally, consider how Partners in Health refused to accept the World Health Organization’s assertion that it was too expensive and difficult to treat drug resistant tuberculosis in poor countries. Partners in Health created a system of community health workers in Lima Peru to provide care for people with multidrug-resistant tuberculosis [39]. Demonstrating that good treatment outcomes were possible, they helped expand care around the world [28].

These stories should inspire solidarity in our global fight against COVID-19. Currently the largest component of the global response effort is through the WHO-led Access to COVID-19 Tools Accelerator or ACT-A.The ACT-A invests in diagnostics, therapeutics and vaccines and supports basic health systems and access [40]. But, even its best funded initiative to support vaccination efforts - COVAX - has struggled to secure the requisite funding to help vaccinate 20% of the global population [41, 42]. Solidarity requires much more. We must do whatever we can to vaccinate the global population without sacrificing other things that are more important. We must also invest significantly in the other aspects of our global response.

Moreover, solidarity requires preparing for, and responding to, many other pandemic and epidemic diseases beyond COVID-19 [43, 44]. COVID-19 has interrupted service delivery for many other terrible diseases from malaria and tuberculosis to a host of neglected tropical diseases around the world. So, we must address the indirect as well as direct effects of the pandemic to save lives and help the most vulnerable amongst us.

We should also address some of the fundamental barriers constraining access to essential medicines and other technologies - including pharmaceutical companies incentives for research and development. Solidarity requires implementing measures to address artificially created legal scarcity. For instance, countries should revisit patenting and licensing laws so that everyone can access new medical technologies at a reasonable cost and in a timely manner [45–47]. New proposals include tying rewards for new innovations, through COVAX or similar mechanisms, to the health impacts of resulting technologies [28, 45]. Countries should then require open access to the patents and other intellectual property constraining access to promote low cost generic production.

Some charge that we should not reduce intellectual property barriers to accessing new innovations because the problem is really manufacturing capacity [48, 49], but we should both rethink incentives for research and development *and* increase manufacturing capacity in poor countries. More generally, we should put basic health systems in place for all [50]. Many poor countries lack the resources, equipment, cold chains, transportation and health infrastructure, and workers to help everyone access essential medicines in a timely manner [51]. Even reliable electricity, clean water, and adequate roads are problems in many locations and putting this basic health system in place will not only allow us to address COVID but many other terrible pandemics that are also ravaging the earth [52]. Solidarity requires protecting the most vulnerable (as well as the rest of us) by making these investments now.

### Conclusion

Our definition of solidarity derived from the African frameworks is, broadly, this: *a sympathetic and imaginative enactment of collaborative gestures and measures to enhance our intrinsic or acquired relatedness so that together we fare well enough.* Embracing it in our efforts to combat COVID-19 requires exercising our imagination to help people everywhere out of concern for the relationships we stand in to others around the world. Doing so requires addressing artificially created (as well as natural) scarcity and reconsidering how we incentivise new research and development as well as putting basic health systems in place to help all. We must also invest significantly in basic health systems and other aspects of wise pandemic preparedness and response. Only by standing in solidarity with others can we together realize our human potential.

## Data Availability

Not applicable.

## Footnotes

1 A thank you to our colleagues at the Independent Resource Group for Global Health Justice, Oxford and Kings College, Noa Mizrachi, and the Global Health Impact Team for incredibly helpful comments and discussion.

2 We do not discuss John Paul *Sartre and Edmund Husserl’s conceptions of solidarity because this would require going to great lengths to explain why the phenomenological approach, with its starting point as the object that appears to consciousness, differs from the approach the African authors we draw on take.*

3 See: Amartya Sen, Reasoning and Disagreement, Isaiah Berlin Lecture, 2011. Oxford Podcasts: https://itunes.apple.com/us/itunes-u/the-isaiah-berlin-lecture/id381700653

4 For more on what faring well enough together might require see [26, 27].
